# The *Yersinia* High-Pathogenicity Island Encodes a Siderophore-Dependent Copper Response System in Uropathogenic Escherichia coli

**DOI:** 10.1128/mBio.02391-21

**Published:** 2022-01-04

**Authors:** George L. Katumba, Hung Tran, Jeffrey P. Henderson

**Affiliations:** a Center for Women’s Infectious Disease Research, Division of Infectious Diseases, Department of Internal Medicine, Washington University School of Medicine, St. Louis, Missouri, USA; National Cancer Institute

**Keywords:** copper-yersiniabactin, *Escherichia coli*, siderophore, transcription

## Abstract

Siderophores are iron chelators used by microbes to bind and acquire iron, which, once in the cell, inhibits siderophore production through feedback repression mediated by the ferric uptake repressor (Fur). Yersiniabactin (Ybt), a siderophore associated with enhanced pathogenic potential among *Enterobacteriaceae*, also binds copper ions during human and experimental murine infections. In contrast to iron, we found that extracellular copper ions rapidly and selectively stimulate Ybt production in extraintestinal pathogenic Escherichia coli. The stimulatory pathway requires formation of an extracellular copper-Ybt (Cu(II)-Ybt) complex, internalization of Cu(II)-Ybt entry through the canonical TonB-dependent outer membrane transporter, and Fur-independent transcriptional regulation by the specialized transcription factor YbtA. Dual regulation by iron and copper is consistent with a multifunctional metallophore role for Ybt. Feed-forward regulation is typical of stress responses, implicating Ybt in prevention of, or response to, copper stress during infection pathogenesis.

## INTRODUCTION

Escherichia coli is the predominant cause of human urinary tract infections (UTIs) (1–3) and, together with other *Enterobacteriaceae*, accounts for a substantial proportion of the worldwide rise in antibiotic-resistant infections (3–5). Although these bacterial species commonly colonize the gastrointestinal tract, their presence in the urinary tract is associated with transcriptional activation of nonessential variable genes in the genome or on plasmids (6–9). Prominent among these are iron-responsive operons encoding iron acquisition systems expressed during human UTIs and experimental animal models of UTI (10).

Multiple siderophore systems, which use secreted, small-molecule chelators to form stable iron(III)-siderophore complexes that are selectively imported by dedicated transport machinery, have been associated with greater pathogenic potential in *Enterobacteriaceae*. Siderophore production in these bacteria is transcriptionally regulated by the ferric uptake repressor (Fur), a global regulator of iron acquisition systems in many microbes (11–14). When cellular iron is abundant, Fur reversibly binds an iron-sulfur cluster and assumes a conformation that binds regulatory sites in the genome, known as Fur boxes, to repress transcription (13–15). The resulting negative feedback circuit allows microbes to avoid the metabolic costs associated with siderophore production when iron is abundant (16). Accordingly, the presence of a Fur box upstream of biosynthetic gene clusters is often used to identify putative siderophore genes in microbial genomes (17).

While all E. coli secrete the genetically conserved siderophore enterobactin, extraintestinal E. coli isolates typically secrete additional siderophores such as yersiniabactin, salmochelin, and aerobactin (6, 8). Multiple gains of function have been proposed to explain why these strains tolerate the metabolic costs of these additional siderophore systems (18–20). The yersiniabactin (Ybt) system is an especially prominent nonconserved siderophore system in urinary E. coli isolates and a conserved siderophore system in pandemic Yersinia pestis. Although initially appreciated for its iron acquisition function, Ybt has recently been observed to form stable complexes with copper, which are detectable in urine specimens from human and murine UTI (10, 21–24). The ability to biosynthesize Ybt was determined to promote intracellular bacterial survival in macrophage-like RAW264.7 cells in a copper and respiratory burst-dependent manner (21). Furthermore, the inner membrane Ybt transporters, YbtPQ, were demonstrated to enhance E. coli fitness during high-titer cystitis using a murine model (25).

Interactions between copper ions and the Ybt system may involve nutritional copper uptake (22) and a protective response to host antibacterial defenses based on copper toxicity (10, 21, 26), functions that are not mutually exclusive. By locking host-derived copper ions into stable complexes, Ybt may shield bacteria from host-derived copper toxicity while enabling continued nutritional access to copper, a microbial strategy termed nutritional passivation (21, 22, 27). Precisely how Cu(II)-Ybt is trafficked within the cell after import from the extracellular space remains unclear. Previous work has provided evidence supporting that bacteria transport intact Cu(II)-Ybt complexes through the outer membrane via FyuA into the periplasm. The inner membrane ABC transporter heterodimer, YbtPQ, was determined to be necessary to reductively yield nutritional copper and metal-free Ybt following import by FyuA (22). Subcellular localization of the reductase and Cu(II)-Ybt to the cytoplasm or periplasm has not been directly assessed.

Despite these nonferric metal ion interactions, iron has been the only transition metal recognized to regulate the Ybt system. Canonical Fur-mediated iron repression, however, appears inadequate to respond to immune responses that increase copper ion concentrations. Here, we used quantitative mass spectrometry, reverse genetics, and transcriptional reporters to compare the effects of copper and iron on Ybt secretion in a model uropathogenic E. coli strain. We found that copper and iron exert distinctive, opposing effects on Ybt secretion. Copper-stimulated Ybt production was transcriptionally mediated and independent of Fur and canonical E. coli copper sensors. The stimulatory signal requires Cu(II)-Ybt formation, is facilitated by the outer membrane importer FyuA, and requires the dedicated transcription factor YbtA, which is predicted to possess a regulatory ligand binding domain characteristic of other AraC-type transcription factors. Thus, free copper ions elicit a copper-triggered positive feedback regulation cycle in which Ybt stimulates its own production.

## RESULTS

### Copper and iron ions have opposing effects on Ybt.

**FIG 1:**
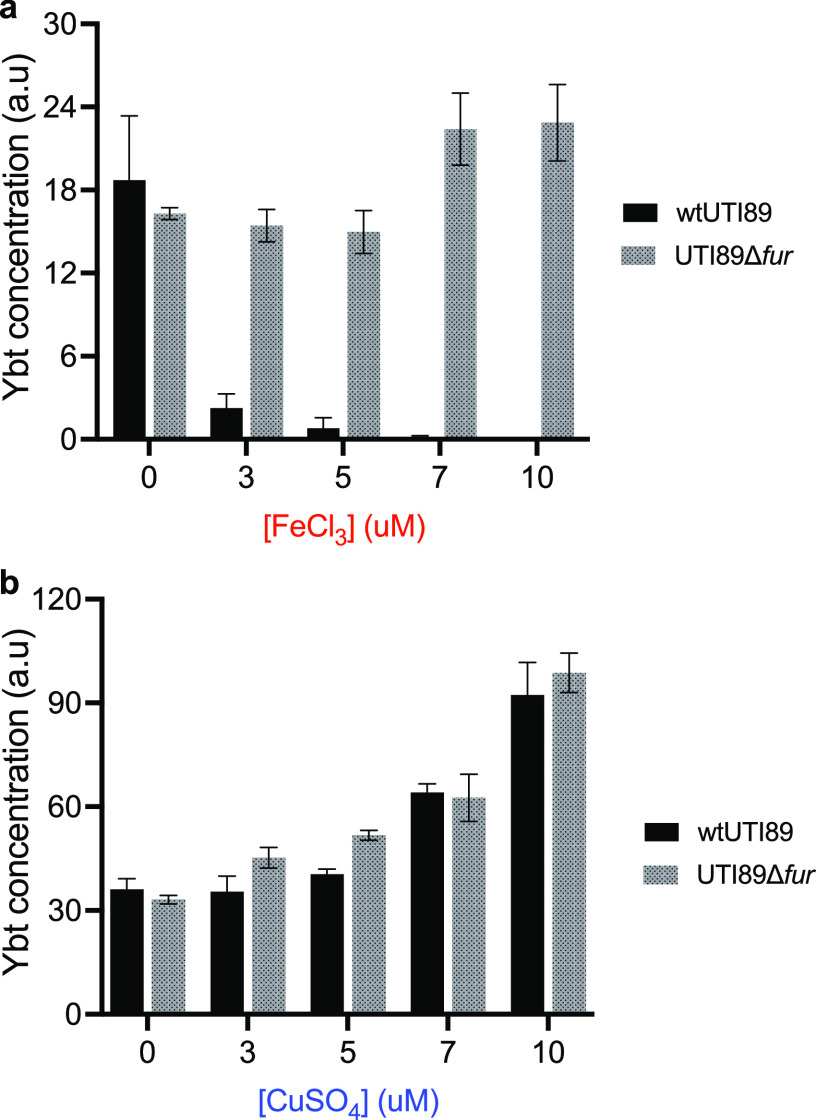
Iron and copper ions exhibit opposing effects on Ybt production. (a) As FeCl_3_ concentrations increase, Ybt levels in UTI89 culture supernatants (black bars) decrease. This relationship is abolished in the UTI89*Δfur* mutant (gray bars). (b) As CuSO_4_ levels increase, Ybt levels in both UTI89 (black bars) and UTI89*Δfur* (gray bars) culture supernatants increase. Data are representative of multiple experiments and plotted as mean peak area ratio ± SD from triplicate determinations.

To compare the effects of copper and iron on Ybt production, we exposed the model uropathogenic E. coli (UPEC) strain UTI89 to increasing concentrations of iron and copper ions in standard M63-glycerol minimal medium. We then quantified Ybt in culture supernatants using a previously described (28) liquid chromatography-tandem mass spectrometry (LC-MS/MS) assay. Typical of siderophore systems, addition of iron to UTI89 cultures was followed by decreased Ybt levels in culture supernatants (Fig. 1a). In contrast, copper addition was associated with increased Ybt levels in culture media, nearly 4-fold higher for 10 μM Cu(II) ions than baseline production (Fig. 1b). The different metal ion responses were not attributable to bacterial density, which was higher in iron-treated cultures (see Fig. S1 in the supplemental material). These results are consistent with a stimulatory effect of copper on Ybt secretion by UTI89.

10.1128/mBio.02391-21.1 FIG S1 Impact of CuSO_4_ and FeCl_3_ addition on bacterial stationary-phase densities. Compared to untreated cultures, addition of Fe(III) increased the stationary-phase bacterial densities of both UTI89 (a) and UTI89*Δfur* (b) strains, attributable to a ready supply of Fe(III). Higher concentrations of Fe(III) did not confer any growth advantage compared to lower Fe(III) concentrations. Addition of Cu(II) did not have any significant effect on stationary-phase bacterial densities of both UTI89 (c) and UTI89*Δfur* (d) cultures. Data are plotted as mean ± SD from triplicate determinations. ns, nonsignificant; ***, *P* = 0.0001; ****, *P* > 0.0001. Download FIG S1, EPS file, 0.7 MB. Copyright © 2022 Katumba et al. 2022 Katumba et al. https://creativecommons.org/licenses/by/4.0/ This content is distributed under the terms of the Creative Commons Attribution 4.0 International license.

### The copper-associated increase in Ybt is Fur independent.

To determine whether Fur is necessary for the copper-associated increase in Ybt concentrations, we next compared the response to iron or copper ion addition by the UTI89*Δfur* Fur-deficient mutant. Iron addition was no longer associated with diminished Ybt concentrations (Fig. 1a) in UTI89*Δfur*-conditioned supernatants, consistent with loss of canonical Fur-mediated repression. The copper-associated increases in Ybt concentration observed in wild-type UTI89, however, were maintained in the UTI89*Δfur* mutant, again reaching nearly 4-fold the baseline production (Fig. 1b) for 10 μM Cu(II) ions. As with wild-type UTI89, iron, but not copper, increased endpoint bacterial density of the UTI89*Δfur* mutant (Fig. S1). These data show that the stimulatory effect of copper on Ybt secretion in UTI89 can occur independently of Fur, the canonical siderophore regulator in E. coli.

### Cu(II)-Ybt and Fe(III)-Ybt exhibit opposing effects on Ybt production.

**FIG 2:**
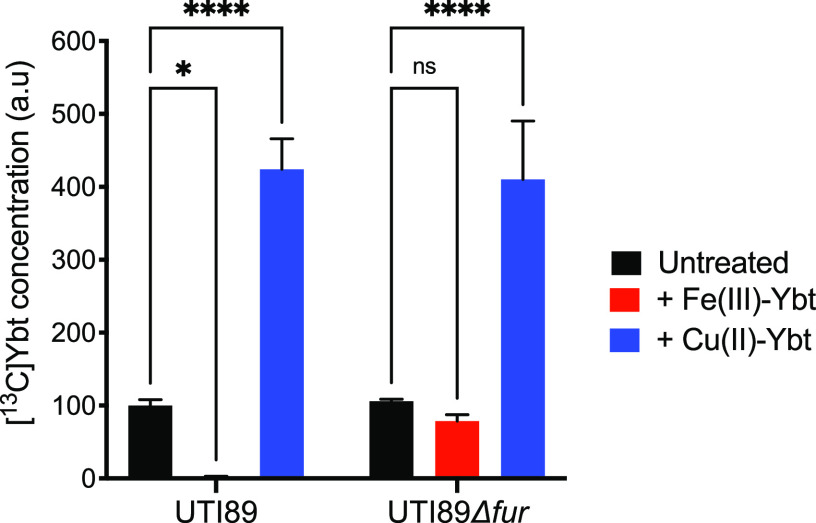
Purified Fe(III)-Ybt and Cu(II)-Ybt exhibit opposing effects on Ybt production. Cultures were grown with a ^13^C-substitute carbon source so that *de novo* Ybt biosynthesis could be distinguished using mass spectrometric quantification. Addition of 3 μM unlabeled Fe(III)-Ybt decreased [^13^C]Ybt concentrations in UTI89 culture supernatants but had an insignificant effect on [^13^C]Ybt in UTI89Δ*fur* supernatants. Addition of 3 μM unlabeled Cu(II)-Ybt increased [^13^C]Ybt concentrations in both UTI89 and UTI89*Δfur* culture supernatants. Data were plotted from a representative experiment as percent mean area ratio ± SD from triplicate determinations. ns, nonsignificant; *, *P = *0.0045; ****, *P < *0.0001 according to Dunnett’s multiple-comparison test.

Ybt spontaneously forms stable 1:1 coordination complexes with labile Cu(II) and Fe(III) ions (23), raising the question of whether metal ions or resulting metal ion-Ybt complexes mediate the effects observed with metal ion additions. To address this question, we compared Ybt production by UTI89 and UTI89*Δfur* strains in response to purified Cu(II)-Ybt or Fe(III)-Ybt. We distinguished newly synthesized Ybt from the purified metal-Ybt reagents by growing strains in culture supplied with [^13^C]glycerol as the metabolic substrate, permitting newly synthesized Ybt to be identified by its ^13^C-associated mass shift. In this experimental system, Fe(III)-Ybt addition was associated with decreased Ybt in cultures of UTI89 but not the UTI89*Δfur* mutant (Fig. 2). Cu(II)-Ybt increased Ybt production by nearly 4-fold in both UTI89 and the UTI89*Δfur* mutant (Fig. 2). Neither change in Ybt production was attributable to differences in stationary-phase bacterial densities (Fig. S2). Together, these results indicate that Cu(II)-Ybt addition is sufficient to stimulate Ybt production in the presence or absence of Fur.

10.1128/mBio.02391-21.2 FIG S2 Addition of Cu(II)-Ybt and Fe(III)-Ybt stimulates bacterial growth. Compared to untreated cultures, addition of Fe(III)-Ybt and Cu(II)-Ybt increased stationary bacterial densities. The increases were attributed to a ready supply of these metal ions as Ybt complexes supplied through the *ybt* import system. Data were plotted as mean ± SD from triplicate determinations. **, *P = *0.0087; ***, *P = *0.0001; ****, *P < *0.0001. Download FIG S2, EPS file, 0.09 MB. Copyright © 2022 Katumba et al. 2022 Katumba et al. https://creativecommons.org/licenses/by/4.0/ This content is distributed under the terms of the Creative Commons Attribution 4.0 International license.

### Copper ions stimulate *ybt* gene transcription.

**FIG 3:**
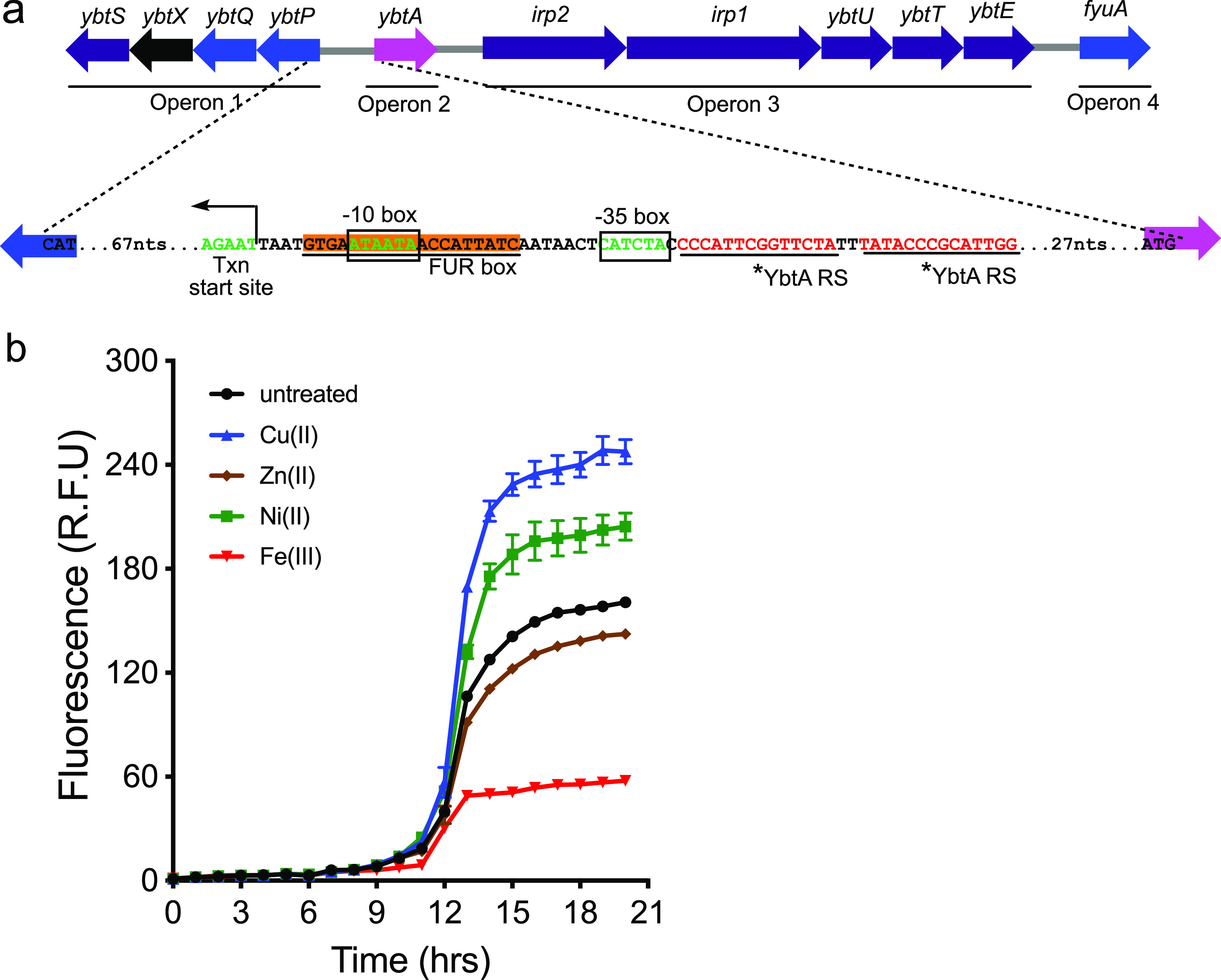
Copper stimulates transcriptional upregulation of the operon containing the first Ybt biosynthetic gene. (a) Gene map of the *Yersinia* high-pathogenicity island in UTI89 showing biosynthesis (purple), transport (blue), and regulatory (magenta) genes and a gene of unknown function (black). The detailed sequence shows the regulatory elements in the promoter for operon 1, which encodes YbtS, the first committed enzyme in Ybt biosynthesis. This promoter was used in the mCherry reporter construct *pGK095*. The FUR box represents the Fur binding site and YbtA RS represents repeat sequences that are putative YbtA binding sites within the promoter region. (b) mCherry fluorescence of UTI89*_pGK095* treated with 3 μM Cu(II), Ni(II), Zn(II), or Fe(III) ions and untreated control (black). R.F.U., relative fluorescence units.

We hypothesized that copper-associated increases in extracellular Ybt occur by transcriptionally upregulating Ybt biosynthesis genes. To test this, we created an mCherry reporter plasmid (*pGK095*) using the *Yersinia* high-pathogenicity island (HPI) operon 1 promoter (Fig. 3a), which encodes YbtS, the first dedicated enzyme in the Ybt biosynthesis pathway, along with three transporters (YbtP, YbtQ, and YbtX). UTI89_*pGK095* exhibited substantially increased mCherry fluorescence following addition of 3 μM Cu(II) ions to the culture medium (Fig. 3b). In contrast, addition of 3 μM Fe(III) ions (Fig. 3b) diminished reporter fluorescence below baseline, consistent with canonical Fur repression. These results are consistent with transcriptional upregulation as the mechanism of copper-mediated increases in Ybt biosynthesis. To further probe divalent metal ion specificity, we assessed the effect of nickel and zinc exposure. Ni(II) ions increased mCherry fluorescence, although not to the same level as that observed with Cu(II) (Fig. 3b). Zn(II) ions had a negligible effect on mCherry fluorescence (Fig. 3b). Thus, these biometals were associated with three distinctive transcriptional responses: stimulation by Cu(II) and Ni(II), repression by Fe(III), and a minimal effect by Zn(II). With the exception of Fe(III), which stimulated stationary-phase bacterial densities due to nutritional growth stimulation, none of the metals affected bacterial growth at the concentrations used in this experiment (Fig. S3a).

10.1128/mBio.02391-21.3 FIG S3 Bacterial growth kinetics are not affected by metal ion or metal-Ybt additions. Growth controls for the strains used to monitor reporter activity in the experiments described in the legend to Fig. S2. (a) Compared to untreated cultures, addition of 3 μM different metal ions did not affect bacteria growth kinetics, except for Fe(III), which slightly increased stationary-phase density. In panels b to f, growth curves of the different strains show that compared to untreated cultures, addition of 3 μM Cu(II)-Ybt did not affect bacteria growth kinetics. Addition of 3 μM Fe(III)-Ybt slightly increased stationary-phase density, consistent with iron-stimulated growth from a ready supply of the metal ion. Data are plotted as mean ± SD from duplicate determinations. Download FIG S3, EPS file, 0.6 MB. Copyright © 2022 Katumba et al. 2022 Katumba et al. https://creativecommons.org/licenses/by/4.0/ This content is distributed under the terms of the Creative Commons Attribution 4.0 International license.

### Ybt is necessary for copper-associated transcriptional activation of *ybt* genes.

**FIG 4:**
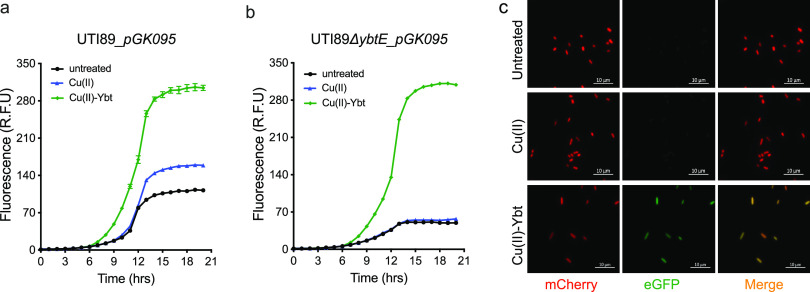
Ybt is necessary for copper-associated transcriptional upregulation. mCherry reporter fluorescence from UTI89*_pGK095* (a) and its UTI89*ΔybtE_pGK095* Ybt-deficient mutant (b) in untreated medium (black) or following addition of 3 μM Cu(II) (blue) or 3 μM Cu(II)-Ybt (green). (c) Fluorescence micrographs of UTI89Δ*ybtE*_*pGK084* mutant carrying a dual reporter with constitutive mCherry and inducible GFP. No GFP fluorescence was detected from the untreated cells detected by mCherry fluorescence; 3 μM Cu(II) addition did not activate GFP fluorescence; and 3 μM Cu(II)-Ybt addition resulted in robust and widespread GFP signal in mCherry-positive cells.

Ybt is both a product and, upon forming the Cu(II)-Ybt complex, a plausible stimulus of Ybt production. To assess this relationship, we used the transcriptional reporter assay, which permits Ybt biosynthetic gene transcriptional regulation to be investigated in Ybt-null mutants. To determine whether Cu(II)-Ybt formation is necessary for copper stimulation of Ybt biosynthesis, we monitored *pGK095* reporter activity in the UTI89Δ*ybtE* strain, a Ybt-deficient mutant lacking the AMP ligase necessary for the second committed step of Ybt biosynthesis (29). In UTI89_*pGK095*, both copper ions and purified Cu(II)-Ybt stimulated reporter fluorescence, consistent with the above-described results (Fig. 4a). In contrast, addition of copper ions alone failed to stimulate reporter fluorescence by the UTI89Δ*ybtE*_*pGK095* mutant. The UTI89Δ*ybtE*_*pGK095* reporter fluorescence response to Cu(II)-Ybt, however, remained intact (Fig. 4b). Fluorescence micrographs of the UTI89Δ*ybtE* mutant carrying a dual reporter construct (*pGK084*) that constitutively expresses mCherry and expresses green fluorescent protein (GFP) under the control of the operon 1 promoter demonstrated widespread GFP fluorescence following treatment with purified Cu(II)-Ybt but not Cu(II) ions alone (Fig. 4c). Together, these results are consistent with Cu(II)-Ybt formation from Cu(II) and Ybt as a necessary step in copper-stimulated transcriptional upregulation of *ybt* genes. Addition of both Cu(II)-Ybt and Fe(III)-Ybt exhibited a noticeable increase in bacterial stationary-phase density owing to a ready supply of the metal ions (Fig. S3b).

### Cu(II)-Ybt elicits a rapid transcriptional response from UTI89 cells.

**FIG 5:**
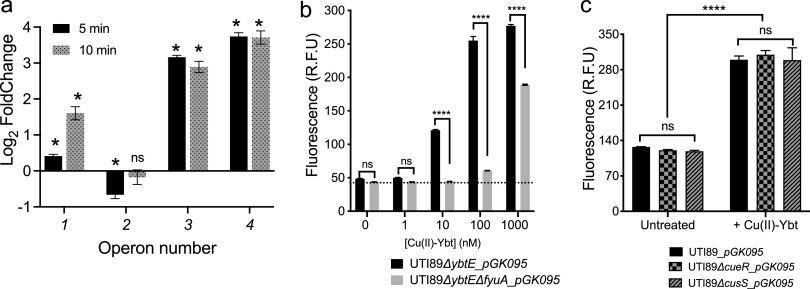
Rapid transcriptional signaling by Cu(II)-Ybt is facilitated by the outer membrane transporter FyuA but not canonical copper sensor systems. mRNA of representative genes from three of the four HPI operons in the UTI89*ΔybtE* mutant measured by qRT-PCR increased significantly after exposure to 5 μM purified Cu(II)-Ybt for 5 min and 10 min. (b) mCherry fluorescence from UTI89*ΔybtE_pGK095* and UTI89*ΔfyuAΔybtE_pGK095* cultures grown for 20 h after addition of increasing concentrations of Cu(II)-Ybt. Deletion of the outer membrane transporter, FyuA, significantly diminishes the reporter response to Cu(II)-Ybt. (c) mCherry fluorescence from untreated and Cu(II)-Ybt-treated (3 μM) cultures of wild-type UTI89_*pGK095* and UTI89*ΔcueR_pGK095* and UTI89Δ*cusS_pGK095* strains. Results indicate that canonical copper-sensing proteins CueR and CusS are not necessary for Cu(II)-Ybt signaling. Data were plotted as mean ± SD from triplicate determinations. ns, nonsignificant; *, *P* < 0.0001 in panel a; ****, *P* < 0.0001 in panels b and c according to Dunnett’s multiple-comparison test.

Transcriptional responses to Cu(II)-Ybt may derive from direct transcription factor activation or from indirect responses to large-scale changes in cellular physiology. To distinguish among these possibilities, we measured transcription in Ybt-deficient UTI89 (UTI89Δ*ybtE* mutant) of the four HPI operons after 5 and 10 min of exposure to purified Cu(II)-Ybt. HPI operon 1, 3, and 4 genes exhibited significant transcriptional activation after 5 and 10 min of exposure to Cu(II)-Ybt (Fig. 5a). Operon 2 mRNA levels were significantly diminished 5 min after Cu(II)-Ybt exposure but exhibited no significant change after 10 min. These rapid transcriptional responses are consistent with a direct mode of transcriptional activation by Cu(II)-Ybt that does not require extensive translation of new proteins. Rapid transcriptional changes such as these are typical of responses to nutritional or cytotoxic stress-associated signals.

### FyuA delivers extracellular Cu(II)-Ybt for signaling.

Gram-negative bacteria typically interact with extracellular ferric siderophore complexes through TonB-dependent transporters (TBDTs) in the outer membrane. FyuA, the TBDT encoded by operon 4 of the *Yersinia* HPI, has been functionally characterized as an outer membrane importer of metal-yersiniabactin complexes in previous work (23, 30, 31). To determine whether FyuA plays a role in the transcriptional response to extracellular Cu(II)-Ybt, we compared *pGK095* reporter activity in the UTI89*ΔybtE_pGK095* mutant to its UTI89*ΔfyuAΔybtE_pGK095* isogenic FyuA-deficient mutant (32). Compared to the UTI89*ΔybtE_pGK095* mutant, the reporter signal from the UTI89*ΔfyuAΔybtE_pGK095* mutant was nonresponsive to 10 nM Cu(II)-Ybt (Fig. 5b and Fig. S4a and b). At Cu(II)-Ybt concentrations of 100 nM or higher, an increased reporter signal is resolved in the UTI89*ΔfyuAΔybtE_pGK095* mutant, although it remains significantly lower than that observed from the UTI89*ΔybtE_pGK095* mutant. This relationship is consistent with FyuA-mediated import of Cu(II)-Ybt at high concentrations that is circumvented by nonspecific, passive outer membrane transport, possibly involving porins. While TBDTs such as FecA activate signaling cascades independently of transport (33), preserved signaling in the FyuA-null mutant is inconsistent with this signaling mechanism in the copper response. Both strains exhibited similar growth dynamics with and without Cu(II)-Ybt addition (Fig. S4c and d). These data, together with the previously reported ability of FyuA to import Cu(II)-Ybt (22, 25), are consistent with FyuA-mediated import of extracellular Cu(II)-Ybt as an important component of the transcriptional response to copper.

10.1128/mBio.02391-21.4 FIG S4 FyuA-mediated import of Cu(II)-Ybt is important for signaling upregulation of *ybt* transcription. (a) Addition of increasing concentrations of Cu(II)-Ybt to UTI89*ΔybtE-pGK095* cultures increases the mCherry reporter signal in a concentration-dependent manner. (b) Addition of equivalent concentrations of Cu(II)-Ybt to UTI89*ΔfyuAΔybtE-pGK095* culture results in lower reporter signal compared to UTI89*ΔybtE-pGK095* culture, indicating reduced sensitivity to Cu(II)-Ybt due to deletion of FyuA. Growth curves of UT89*ΔybtE_pGK095* (c) and UTI89*ΔfyuAΔybtE_pGK095* (d) cultures show that addition of Cu(II) ions or Cu(II)-Ybt at the specified concentrations does not affect the growth dynamics of the bacterial cultures. Download FIG S4, EPS file, 0.4 MB. Copyright © 2022 Katumba et al. 2022 Katumba et al. https://creativecommons.org/licenses/by/4.0/ This content is distributed under the terms of the Creative Commons Attribution 4.0 International license.

### Cu(II)-Ybt stimulates *ybt* gene transcription independently of canonical copper sensors.

Although the specific mechanisms of intracellular metal-Ybt trafficking remain unclear, previous work has found that copper can be removed from Cu(II)-Ybt following FyuA-mediated import (22). We therefore hypothesized that Cu(II)-Ybt-derived copper stimulates HPI operon 1 transcription through one of the canonical copper sensors. To test this, we assessed *pGK095* reporter activity in defined copper-sensing mutants. Two copper-specific response systems have been described in E. coli: the MerR-family transcriptional factor CueR, which directly responds to cytoplasmic Cu(I) ions (32, 34, 35), and the CusRS two-component system, which senses and responds to periplasmic Cu(I) ions (36–39). We observed that Cu(II)-Ybt stimulated reporter activity in both UTI89*ΔcueR*_*pGK095* and UTI89*ΔcusS*_*pGK095* strains to levels similar to those of wild-type UTI89_*pGK095* (Fig. 5c, Fig. S5a and b). Baseline reporter activity from all three untreated strains was comparable. Addition of Cu(II)-Ybt did not impact the growth dynamics of any of the cultures relative to untreated cultures (Fig. S5c and d). These results rule out a role for canonical E. coli copper sensors in Cu(II)-Ybt-associated transcriptional upregulation and are consistent with a noncanonical mode of copper sensing in UTI89.

10.1128/mBio.02391-21.5 FIG S5 Canonical E. coli copper sensors are not required for Cu(II)-Ybt signaling. UTI89*ΔcueR_pGK095* (a) and UTI89*ΔcusS_pGK095* (b) cultures exhibit similar reporter activities and responses to Cu(II)-Ybt as wild-type UTI89_*pGK095*. Addition of Cu(II)-Ybt did not affect growth kinetics of UTI89_pGK095, UTI89*ΔcueR_pGK095* (c), and UTI89*ΔcusS_pGK095* (d) cultures compared to their respective untreated cultures. Data are plotted as mean ± SD from triplicate determinations. Download FIG S5, EPS file, 0.9 MB. Copyright © 2022 Katumba et al. 2022 Katumba et al. https://creativecommons.org/licenses/by/4.0/ This content is distributed under the terms of the Creative Commons Attribution 4.0 International license.

### YbtA is required for stimulation of operon 1 transcription by Cu(II)-Ybt.

**FIG 6:**
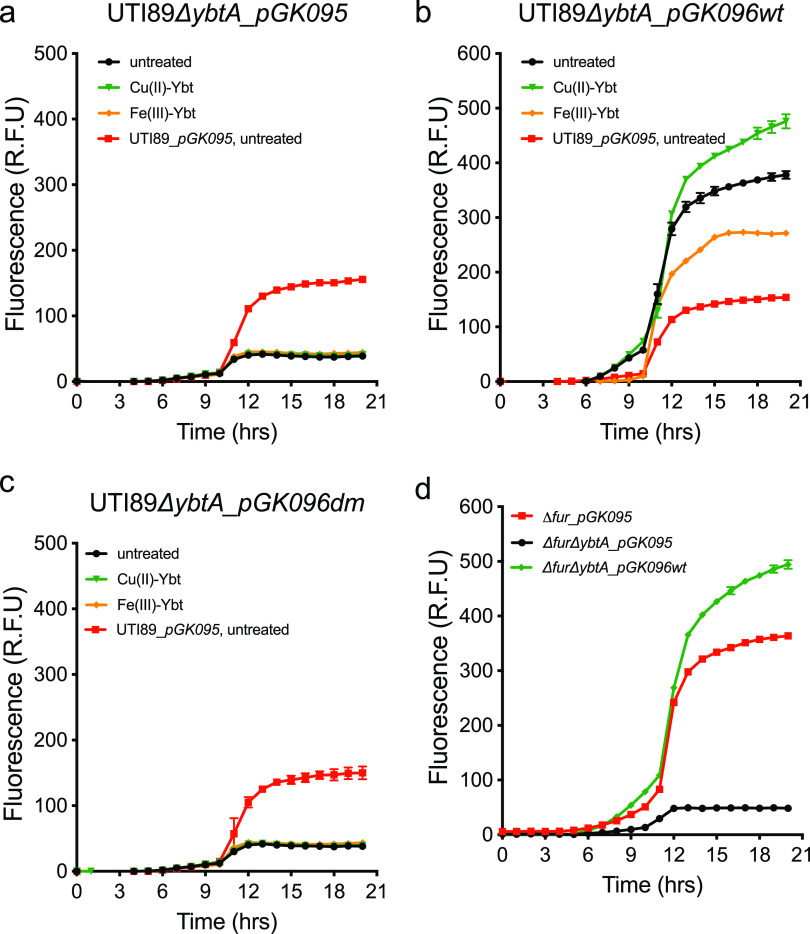
YbtA is necessary for activation of *ybt* gene transcription. (a) The YbtA-deficient UTI89*ΔybtA_pGK095* mutant exhibits minimal fluorescence that is unresponsive to 3 μM Cu(II)-Ybt (green) or 3 μM Fe(III)-Ybt (orange). Untreated wild-type UTI89_*pGK095* (red) is included as a control. (b) Genetic complementation of *ybtA* in the UTI89*ΔybtA_pGK096wt* strain restores reporter responses to Cu(II)-Ybt and Fe(III)-Ybt. (c) Genetic complementation with *ybtA* containing two cysteine point mutations (C206S and C209S) with the UTI89*ΔybtA_pGK096dm* strain did not restore responses, consistent with a role in YbtA activity. (d) The UTI89*ΔfurΔybtA_pGK095* YbtA- and Fur-deficient mutant exhibits minimal fluorescence in the presence of 3 μM Cu(II)-Ybt (black) compared to the UTI89*Δfur_pGK095* Fur-deficient mutant (red). Genetic complementation of *ybtA* in the UTI89*ΔfurΔybtA*_*pGK096wt* strain restored the reporter response to 3 μM Cu(II)-Ybt (green). Data were plotted as mean ± SD from triplicate experiments.

Without a necessary role for Fur, CueR, and the CusRS system, we assessed the role of the *Yersinia* HPI-encoded transcription factor, YbtA, the function of which was previously investigated in the context of iron responses (40–43). To determine whether YbtA is necessary for Cu(II)-Ybt-associated transcriptional upregulation, we monitored *pGK095* reporter activity in a *ybtA* deletion mutant. Relative to wild-type UTI89_*pGK095*, reporter activity from the UTI89*ΔybtA*_*pGK095* mutant was minimal in both the presence and absence of Cu(II)-Ybt addition (Fig. 6a). Reporter activity was genetically complemented by ectopic expression of *ybtA* on plasmid *pGK096w*t (Fig. 6b, Fig. S6c). Of note, reporter activity in the UTI89*ΔybtA*_*pGK096wt* mutant exceeded that of the UTI89_*pGK095* strain, consistent with a gene dosage effect. As observed in the UTI89_*pGK095* strain, Cu(II)-Ybt and Fe(III)-Ybt increased and decreased fluorescence, respectively, in the UTI89*ΔybtA*_*pGK096wt* mutant (Fig. 6b) compared to the untreated baseline of the UTI89*ΔybtA*_*pGK096wt* mutant. The concentrations of copper and Cu(II)-Ybt used in these experiments did not affect bacterial growth (Fig. S3b, d, and e). These data demonstrate that *ybtA* is necessary for copper-stimulated *ybt* gene transcription.

10.1128/mBio.02391-21.6 FIG S6 Protein binding sites and sequence maps. (a) Alignment of upstream HPI promoter sequences showing Fur binding sites and pseudopalindromic sequences that are potential YbtA binding sites. (b) Domain map of YbtA showing the predicted ligand (purple) and DNA (green) binding domains. YbtA contains 11 cysteines located primarily in the predicted ligand binding domain and the interdomain linker. Residues that were mutated (C206S and C209S) are shown in red. (c) Plasmid map of the reporter construct used to complement *ybtA*. The mCherry reporter is under the control of the operon 1 promoter, and *ybtA* is expressed under the control of its native promoter. Download FIG S6, TIF file, 0.4 MB. Copyright © 2022 Katumba et al. 2022 Katumba et al. https://creativecommons.org/licenses/by/4.0/ This content is distributed under the terms of the Creative Commons Attribution 4.0 International license.

### YbtA is required for operon 1 transcription even in the absence of Fur repression.

To determine if loss of Fur repression eliminated the YbtA requirement for transcription, we monitored the reporter activity from the UTI89*ΔfurΔybtA_pGK095* mutant in the presence of Cu(II)-Ybt. While robust reporter activity was detected from the UTI89*Δfur*_*pGK095* mutant, minimal mCherry fluorescence was detected from the UTI89*ΔfurΔybtA*_*pGK095* mutant (Fig. 6d). Genetic complementation with ectopic *ybtA* expression on plasmid pGK096wt restored reporter activity in the double deletion mutant (Fig. 6d). These data provided further evidence that YbtA is not simply required as a Fur derepression mechanism but rather plays a critical role in activating *ybt* gene transcription independently of Fur repression. All strains used in this experiment exhibited similar growth kinetics in the presence of Cu(II)-Ybt (Fig. S3c).

### Residues C206 and C209 are critical for YbtA activity.

As an AraC family transcription factor, YbtA contains a predicted C-terminal DNA binding domain (DBD) as well as a distinct N-terminal ligand binding domain (LBD) (Fig. S6b) that typically binds small-molecule regulators (44). YbtA’s predicted LBD is notably cysteine rich, with 11 cysteine residues, and includes a CXXC motif that is typically found in copper-binding proteins (45). Structurally conservative cysteine-to-serine substitutions of this CXXC motif, C206S and C209S, abolished mCherry fluorescence from the UTI89*ΔybtA*_*pGK096dm* mutant (where *dm* indicates double point mutation) (Fig. 6c). These results are consistent with a determinative role for specific structural features in the N-terminal LBD in YbtA-dependent transcriptional upregulation of HPI genes. The addition of Cu(II)-Ybt or Fe(III)-Ybt did not affect bacterial growth at these concentrations (Fig. S3f).

## DISCUSSION

The opposing responses to iron and copper by the Ybt system are attributable to a previously unappreciated copper response functionality that operates alongside a canonical siderophore activity. The copper response requires Ybt to act as an extracellular copper recognition element that enters the cell through FyuA and activates transcription independently of canonical E. coli copper-sensing systems. Copper-dependent transcriptional activation is achieved independently of the Fur derepression typical of siderophore systems and is dependent upon YbtA, a transcription factor encoded by the *Yersinia* high-pathogenicity island. The role of Ybt as both stimulus [as Cu(II)-Ybt] and response (through increased biosynthesis) is a form of feed-forward regulation that is found among bacterial stress response systems. Its presence in the *Yersinia* HPI implicates extracellular copper responses in *Enterobacteriales* with increased pathogenic potential.

**FIG 7:**
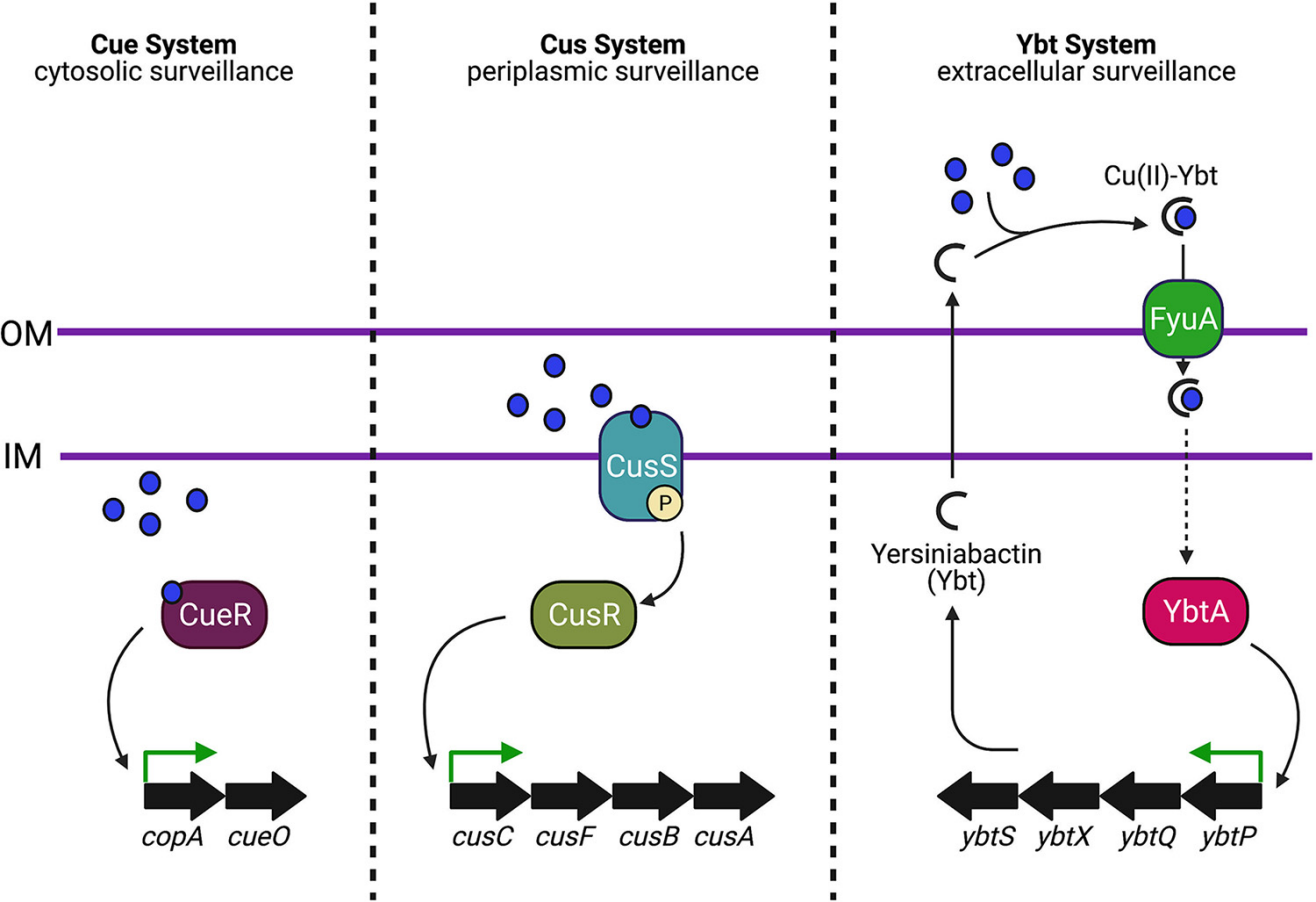
Model for tricompartmental copper ion detection in uropathogenic E. coli. The Cue and Cus systems maintain copper homeostasis in E. coli by sensing and responding to copper ions in the cytoplasm and periplasm, respectively. Data in this report are consistent with detection and response to extracellular copper ions using secreted yersiniabactin (Ybt) acting as both a soluble receptor and a copper-binding effector. The sensing pathway requires formation of stable Cu(II)-Ybt complexes, import through the outer membrane transporter, FyuA, and a YbtA-dependent signaling process that upregulates Ybt biosynthetic genes.

Our results point to a mode of extracellular copper sensing that is independent of the two canonical E. coli copper response systems, CusRS and CueR, that detect intracellular copper in the periplasm and cytoplasm, respectively. These systems are schematically represented in our working model for E. coli copper sensor-response systems in Fig. 7. Each of these systems transcriptionally activates a copper countermeasure (Ybt or a copper efflux pump) to respond to copper in the subcellular space where it is sensed. Our model also expands our understanding of Ybt-mediated copper import and its role in mitigating cellular copper toxicity. By importing Cu(II)-Ybt as a source of nutritional copper, bacteria take up a form of copper whose reactivity is passivated through its interaction with the Ybt molecule. Cu(II)-Ybt is then transported through the Ybt transport pathway, which enables bacteria to control where and how the copper is trafficked within the cell, limiting the cytotoxicity of intracellular labile copper ions.

For the Ybt system, activation by extracellular Cu(II)-Ybt provides direct mechanistic feedback on copper sequestration (10), directing the cell to reinforce an effective cytoprotective strategy. The specific mechanisms of metal-Ybt complex trafficking and dissociation that occur after FyuA-mediated import and before YbtA-mediated transcriptional activation remain to be elucidated. It is therefore an open question whether intact Cu(II)-Ybt, a dissociated product thereof, or an additional protein-protein interaction is the intracellular signal leading to YbtA-mediated transcriptional upregulation. Previous studies have shown that metal-Ybt complexes are primarily imported across the outer membrane into the periplasm via FyuA (23, 30, 32). The inner membrane proteins YbtPQ were determined to be necessary for reductive dissociation of the metal ions from the Ybt molecules and subsequent regeneration of metal-free Ybt (22, 24). However, the identity of the reductase and the location of reduction (periplasm or cytoplasm) remains unclear. YbtA, which is localized in the cytoplasm, may therefore directly sense and respond to Cu(II)-Ybt, Ybt and copper ions, or another related signal through direct interaction with its N-terminal domain, which typically constitutes a ligand binding domain in AraC family transcription regulators (AFTRs). In most AFTRs, transcription factor activity is directly regulated by the associated biosynthetic product or environmental cue relevant to the product’s associated function (44, 46–48).

Ybt biosynthetic regulation in this work is consistent with separable inputs from Fur and YbtA. Our results are consistent with traditional Fur-mediated iron repression while also revealing Cu(II)-Ybt-associated activation as mediated by YbtA. This dual control is not without precedent in siderophore systems. For example, expression of pyochelin in P. aeruginosa is controlled both by Fur and a feedback response from the siderophore mediated by the AFTR, PchR (49). In extraintestinal infection environments, results from a wide range of studies are consistent with low iron availability and Fur derepression (50, 51), which likely leads to at least a low level of Ybt production. Copper stimulation of Ybt production is superimposable on this background and, in this study, is observed even in a *fur*-deficient strain. Further biochemical investigations are required to better understand the mechanistic inputs of YbtA and Fur on the dual regulation of the Ybt system.

In Y. enterocolitica, upstream leader sequences of each of the HPI operons contain not only Fur-boxes for Fur repression but also pseudorepeat sequences, depicted as YbtA RS on the schematic in Fig. 3a, that are predicted to be YbtA binding sites (42). Previous studies showed that addition of spent supernatants from Ybt-producing *Yersinia* strains correlated with an increase in operon 1 expression and a decrease in *ybtA* expression (41, 52). This observation is consistent with the operon 1 and 2 transcriptional responses to Cu(II)-Ybt observed in the present study. We speculate that the delay in operon 1 activation relative to operons 3 and 4 in this study reflects the divergent nature of the promoters controlling operon 1 and operon 2 expression. Specifically, the transcription factor engagement of these overlapping promoters is competitive, such that transcription factors must be displaced from the operon 2 promoter to permit maximal operon 1 activation. Although additional studies are necessary to achieve a more precise accounting of how transcription factor activity and promoter region architecture interact to regulate the Ybt system, the present results are consistent with rapid transcriptional upregulation of multiple HPI operons by Cu(II)-Ybt.

Transcriptional studies have demonstrated that Fur-regulated genes, including siderophores, are expressed by bacteria during infection (53, 54). This observation indicates that widespread iron limitation during infection is a baseline condition under which copper toxicity also occurs. For example, the copper importer CTR1 is upregulated in macrophages during infection, followed by copper-stimulated trafficking of ATP7A from the Golgi to the phagolysosome (55, 56). This effectively mobilizes copper to the phagolysosome, where it was demonstrated to kill internalized bacteria (55). Ybt expression correlated with increased bacterial survival and tolerance under these conditions (21). By binding Cu(II) ions, Ybt sequesters the metal ions away from the bacteria and also inhibits their reduction to the more toxic Cu(I) ions. Moreover, the Cu(II)-Ybt complex exhibited superoxide dismutase-like activity that mitigated the oxidative stress from NADPH oxidase-derived superoxide ions (21). Ybt therefore offers a three-pronged copper response that enhances bacterial survival during infection.

Non-iron metal ion regulation has recently been appreciated in a few other microbial metal ion chelator systems. Opine siderophores (57) in Staphylococcus aureus are also subject to dual metal ion repression by iron and zinc, through Fur and Zur activity, respectively. This reflects the ability of opine siderophores to bind and deliver zinc(II) ions to bacteria that produce them (57–59). Environmental methanotrophs secrete methanobactin as part of a prototypical copper scavenging system, which is repressed when copper is abundant (60–62). The Ybt system differs from these metallophores in that it is stimulated, rather than repressed, in the presence of a metal ion ligand (Cu). This difference may reflect an adaptation to metal ion toxicity, rather than nutritional scarcity alone, by the Ybt system. Identification of other metal ion-associated regulatory pathways may identify new metal ion homeostatic functions in other microorganisms.

## MATERIALS AND METHODS

### Bacteria strains and cultures.

Bacteria were routinely cultured in LB overnight at 37°C with continuous shaking. Dense overnight cultures were inoculated into defined M63-glycerol minimal medium. Deletion mutants made in this study were generated using the Red recombinase protocol (63) with plasmid *pKM208* expressing the recombinase protein. Chloramphenicol resistance cassettes were amplified from *pKD3* and their insertion checked using primers flanking the deleted gene after selection on LB plus chloramphenicol (34 μg/ml; Gold Biotechnologies) plates overnight. All the strains used in this study are shown in Table S1 in the supplemental material, and the primers used to make the deletion mutants are shown in Table S2a.

10.1128/mBio.02391-21.7 TABLE S1 Strains used in this study. All strains used in the current study are listed in this table. Genetic UTI89 mutants were generated using the red recombinase method and transformed with the respective reporter plasmids as indicated. Download Table S1, DOCX file, 0.02 MB. Copyright © 2022 Katumba et al. 2022 Katumba et al. https://creativecommons.org/licenses/by/4.0/ This content is distributed under the terms of the Creative Commons Attribution 4.0 International license.

10.1128/mBio.02391-21.8 TABLE S2 Primer sequences used in this study. (a) Primer sequences used to make gene knockouts to generate UTI89 genetic mutants used in this study. (b) Primer sequences that were used to make *pGK095* reporter constructs carrying the mCherry reporter and *pGK084* carrying the GFP and mCherry dual reporter. (c) Primer sequences for the gene-specific primers used for qRT-PCR. Download Table S2, DOCX file, 0.02 MB. Copyright © 2022 Katumba et al. 2022 Katumba et al. https://creativecommons.org/licenses/by/4.0/ This content is distributed under the terms of the Creative Commons Attribution 4.0 International license.

### Ybt extraction.

Bacteria were cultured in M63-glycerol media containing increasing concentrations of either CuSO_4_ or FeCl_3_, as noted in the figure legends. Cultures were incubated at 37°C with continuous shaking for 22 h. Culture supernatants were harvested at the end of the incubation; 5 μl of 1 M CuSO_4_ or 1 M FeCl_3_ was added to the supernatants from cultures containing CuSO_4_ or FeCl_3_, respectively. Leucine-enkephalin (Sigma) was spiked into the cultures as an internal standard, and the mixture was briefly vortexed to mix thoroughly. Mixtures were centrifuged and 500 μl of the supernatant was transferred to respective wells of a 96-well filter plate with 0.45-μm low-binding hydrophilic polytetrafluoroethylene resin (Millipore) and centrifuged. Aliquots of the filtrate were transferred to mass spectrometry vials and loaded onto the liquid chromatography-mass spectrometer for detection and quantification.

### Ybt detection and quantification.

Extracted siderophores were detected and quantified using a Shimadzu ultrafast liquid chromatography-equipped AB-Sciex 4000 QTrap operated in positive ionization mode using a Turbo V electrospray ionization ion source as previously described (22). Briefly, 5 μl of each sample was injected onto a fused-core phenylhexyl column (100 by 2 mm, 2.7-μm particle; Ascentis Express; Supelco) with a flow rate of 0.4 mL per min. The following gradient was used: solvent A (0.1% [vol/vol] formic acid) was held constant at 95% and solvent B (90% [vol/vol] acetonitrile, 0.1% [vol/vol] formic acid) at 5% for 2 min. Solvent B was increased to 65% by 6 min and to 98% by 8 min. Solvent B was then held constant at 98% until 9 min before it was decreased to 5% by 11 min. Solvent B was then held constant at 5% for 1 additional minute. The collision energy was set at 37 V, and the mass analyzers (Q1)/(Q3) were 535.6/348.5 *m/z*, 543.6/356.1 *m/z*, and 556.2/397.1 *m/z* for Fe(III)-Ybt, Cu(II)-Ybt, and L-enkephalin, respectively. The relative amount of Ybt present in culture supernatants was determined as the quotient of the peak areas of the analyte and the internal standard. Data were plotted using GraphPad Prism9 as mean peak area ratio ± SD for *n* = 3.

### Making fluorescent reporter constructs.

Protein expression vector pMAL-c5Xa (NEB) was used as a backbone for constructing the mCherry reporter used for the bulk of the experiments in this study. The *malE* gene and the *lacI* promoter were restricted from the vector using SacI and KasI restriction enzymes. The mCherry gene was amplified with primers GK073-F/GK073-R and inserted into the pMAL-c5Xa backbone. The operon 1 promoter region was then amplified from the UTI89 genome using primers GK074-F/GK074-R and inserted into the construct described above. The resulting plasmid was named pGK074. To enable complementation of *ybtA*, a different reporter construct (pGK095) was made by amplifying the promoter of operon 1 and the mCherry gene, from plasmid pGK074, using primers GK095-F/GK095-R and inserting the fragment into the pMAL-c5Xa backbone restricted with ApaI and HindIII. To make the reporter/complement plasmid, pGK096wt, the *ybtA* gene was amplified using primers GK096-F/GK096-R and inserted into pGK095. Point mutations C206S and C209S were made on this plasmid using primers C206S, C209S-F/C206S, and C209S-R. The reporter constructs were transformed into respective strains as desired. A dual reporter with constitutive mCherry and inducible GFP was made by inserting the operon 1 promoter sequence into plasmid *pFCcGi* (Addgene) upon restriction with HindIII and XbaI (NEB). The resulting construct was named *pGK084*. The reporter construct was transformed into the UTI89*ΔybtE* strain and used for the microscopy experiment described above. The primer sequences used in creating these constructs are shown in Table S2b.

### Quantitative fluorimetry and bacterial growth curves.

Bacteria were cultured in LB at 37°C with continuous shaking overnight. The dense overnight cultures were used to inoculate M63-glycerol medium plus ampicillin (100 μg/ml; Gold Biotechnologies) at a 1:100 dilution factor. Cultures were treated with different additives at the concentrations specified in the figure legends. For each of at least three independent experiments, triplicate 200-μl aliquots of the M63 cultures were applied onto individual wells of a black, flat-bottom, 96-well plate (Corning) and loaded onto a Tecan Spark plate reader. mCherry fluorescence was monitored hourly over a 20-h growth period at 37°C. The excitation and emission wavelengths were 554 nm and 610 nm, respectively. Fluorescence was detected using a Dichroic520 mirror with 30 flashes and an integration time of 40 s. The Z-position was manually set at 20657, and a manual gain of 90 was applied to the detected fluorescence intensity measurements. Identical experiments were set up for bacterial growth controls. Aliquots (200 μl) of cultures treated similarly to those used in the fluorimetry experiments were applied onto individual wells of a clear, flat-bottom, 96-well plate (Corning) and loaded onto the plate reader. Bacterial growth was monitored hourly by measuring the absorbance at 600 nm.

### Fluorescence microscopy.

The Ybt-deficient UTI89*ΔybtE* strain, transformed with the dual reporter construct *pGK084*, was cultured in LB plus ampicillin (100 μg/ml; Gold Biotechnologies) overnight at 37°C. Overnight cultures were used to inoculate M63-glycerol medium supplemented with either 3 μM CuSO_4_ or 3 μM Cu(II)-Ybt. An untreated control culture was also included. M63 cultures were incubated at 37°C for 22 h with continuous shaking. Cultures were harvested at the end of the growth period and washed twice with 1× phosphate-buffered saline. Aliquots were applied onto glass slides for microscopy. Both mCherry and GFP were detected from the cultures. mCherry was detected at excitation and emission wavelengths of 545 nm and 572 nm, respectively. GFP was detected at excitation and emission wavelengths of 489 nm and 509 nm, respectively. Micrographs were captured using a Zeiss Cell Observer inverted microscope with a color camera.

### Determining gene fold change.

The Ybt-deficient UTI89*ΔybtE* strain was cultured in LB at 37°C with continuous shaking overnight. Dense overnight cultures were inoculated into M63-glycerol medium and cultured at 37°C until the optical density was 0.6 to 0.8. Cultures were then treated with 5 μM Cu(II)-Ybt and incubated for 5 and 10 min postexposure before the cells were harvested and total RNA extracted with a kit (Macherey-Nagel) by following the kit manufacturer’s protocol. RNA concentrations were determined and normalized for all extractions. cDNA was synthesized using the SuperScript first-strand synthesis kit (Invitrogen) by following the manufacturer’s protocol. Real-time quantitative PCR (RT-qPCR) reactions were set up with iTaq Universal SYBR green supermix (Bio-Rad) and conducted on a Bio-Rad CFX96 real-time system. The genes *ybtS*, *ybtA*, *irp2*, and *fyuA* were used to assess transcriptional changes for operons 1, 2, 3, and 4, respectively. Fold changes for respective genes were determined using the formula fold change = 2^−ΔΔ^*^CT^*, with *gyrA* as the reference gene. The log_2_(fold change) then was determined and plotted using Prism software. Untreated control experiments were also conducted and processed similarly to determine the fold change after 5 and 10 min. The specific primers used for fragment amplification and threshold cycle determination are listed in Table S2c.

### Identification of potential Fur and YbtA binding sequences.

Intergenic leader sequences upstream of all *Yersinia* HPI operons were aligned using the open-source software Clustal Omega (64). The alignment revealed that all leader sequences contained approximately 17-bp palindromic sequence elements with high sequence similarity to the Fur box consensus sequence. We also determined that these sequences contained another set of pseudopalindromes that we speculate to be YbtA binding sites.
