# Supplementary figures and images for: The Yersinia High-Pathogenicity Island Encodes a Siderophore-Dependent Copper Response System in Uropathogenic Escherichia coli
Source: mBio. 2022 Jan 4;13(1):e02391-21. doi: 10.1128/mBio.02391-21 (PMC8725597; doi:10.1128/mBio.02391-21)

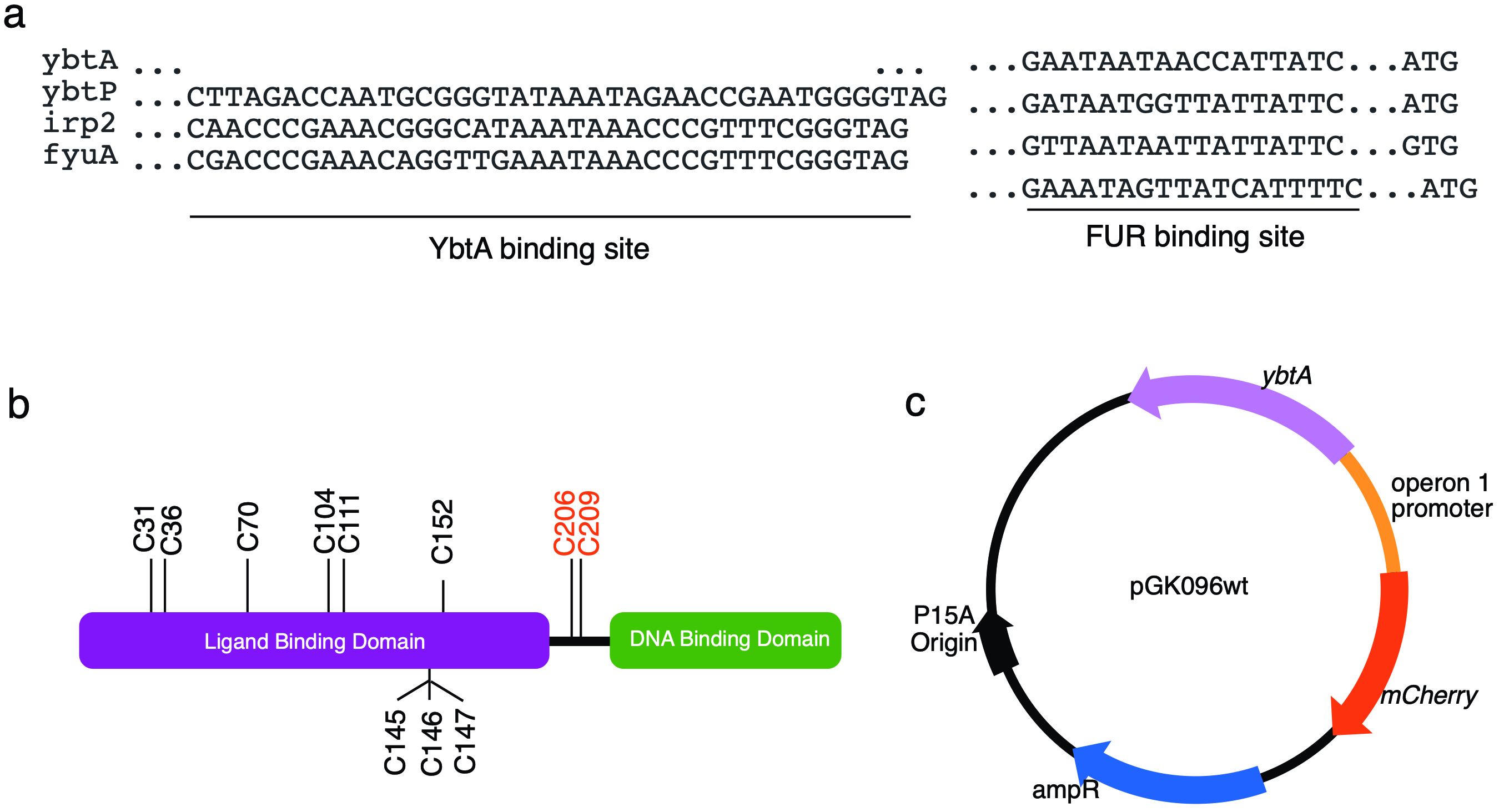

Supplement: FIG S6 [file mbio.02391-21-sf006.tif]
