# Supplementary material for: The Yersinia High-Pathogenicity Island Encodes a Siderophore-Dependent Copper Response System in Uropathogenic Escherichia coli
Source: mBio. 2022 Jan 4;13(1):e02391-21. doi: 10.1128/mBio.02391-21 (PMC8725597; doi:10.1128/mBio.02391-21)
Supplement: TABLE S1 [file mbio.02391-21-st001.docx]

**Table S1: Strains used in this study**

| Strain | Phenotype | Refs |
| --- | --- | --- |
| UTI89 | Clinical isolate of acute cystitis. | ^65^ |
| UTI89*_pGK095* | UTI89 transformed with mCherry reporter plasmid. | This study |
| UTI89*Δfur* | Fur deletion mutant lacking iron-mediated Fur repression. | ^66^ |
| UTI89*Δfur_pGK095* | UTI89*Δfur* transformed with mCherry reporter plasmid. | This study |
| UTI89*ΔybtE* | Ybt-deficient UTI89 deletion mutant. | ^66^ |
| UTI89*ΔybtE_pGK095* | UTI89*ΔybtE* transformed with mCherry reporter plasmid. | This study |
| UTI89*ΔfyuA* | FyuA deletion mutant lacking outer membrane Ybt transporter. | ^66^ |
| UTI89*ΔfyuAΔybtE* | Ybt biosynthesis and transport deficient UTI89 mutant. | This study |
| UTI89*ΔfyuAΔybtE_pGK095* | UTI89*ΔfyuAΔybtE* transformed with mCherry reporter plasmid. | This study |
| UTI89*ΔcueR* | CueR deletion mutant lacking cytosolic copper sensor. | This study |
| UTI89*ΔcueR_pGK095* | UTI89*ΔcueR* transformed with mCherry reporter plasmid. | This study |
| UTI89*ΔcusS* | CusS deletion mutant lacking periplasmic copper sensor. | This study |
| UTI89*ΔcusS_pGK095* | UTI89*ΔcusS* transformed with mCherry reporter plasmid. | This study |
| UTI89*ΔybtA* | YbtA deletion mutant lacking HPI encoded transcription activator. | ^66^ |
| UTI89*ΔybtA_pGK095* | UTI89*ΔybtA* transformed with mCherry reporter plasmid. | This study |
| UTI89*ΔybtA_pGK096wt* | UTI89*ΔybtA* reporter complemented with wildtype *ybtA* gene. | This study |
| UTI89*ΔybtA_pGK096C206S,C209S* | UTI89*ΔybtA* reporter complemented with mutated *ybtA* gene. | This study |
| UTI89*ΔfurΔybtA* | Fur and YbtA double deletion mutant. | This study |
| UTI89*ΔfurΔybtA_pGK095* | UTI89*ΔfurΔybtA* transformed with mCherry reporter plasmid. | This study |
| UTI89*ΔfurΔybtA_pGK096wt* | UTI89*ΔfurΔybtA* reporter complemented with wildtype *ybtA* gene. | This study |
