# Supplementary material for: The Yersinia High-Pathogenicity Island Encodes a Siderophore-Dependent Copper Response System in Uropathogenic Escherichia coli
Source: mBio. 2022 Jan 4;13(1):e02391-21. doi: 10.1128/mBio.02391-21 (PMC8725597; doi:10.1128/mBio.02391-21)
Supplement: TABLE S2 [file mbio.02391-21-st002.docx]

**Table S2**

**a.**

| Gene | Primer ID | Sequence |
| --- | --- | --- |
| *ybtA* | GK094-F | GGTCTAAGCCACAGGGAGATAACCAGGTCATGACGGAGTCACCGCAAACG-GTGTAGGCTGGAGCTGCTTC |
|  | GK094-R | CAGGGAGGAGTTTAGGGGGGCGCGACCCCGGTTACATCCCGCGTTTAAAG- CATATGAATATCCTCCTTAG |
|  | GK094-seqF | CAAAAACGACAATTCAGGGTG |
|  | GK094-seqR | CAATTGAATGCTACCCGAAAC |
| *ybtE* | GK099-F | ATGAATTCTTCCTTTGAATCTCTGATTGAACAGTATCCCTTACCCATTGC-GTGTAGGCTGGAGCTGCTTC |
|  | GK099-R | TATTTCAACCTGTTTCGGGTCGGTTTGCGCTTATTGGGCAGAATGGCGAT-CATATGAATATCCTCCTTAG |
|  | GK098-F | GATCGGATCCATGAATTCTTCCTTTGAATCTCTG |
|  | GK098-R | GATCAAGCTTGCTTATTGGGCAGAATGGC |
| *cueR* | GK079-F | GAATATTTGTTGAAACCCTTTAACAAAGCACAGGAGGCGTTGCGCGAACG-GTGTAGGCTGGAGCTGCTTC |
|  | GK079-R | GTAGAAAGTTTGCAGCATATTCTTTCGGTGCAAACGGAGATTAACTGATT-CATATGAATATCCTCCTTAG |
|  | GK079-seqF | AGTACTTCCTGTATTATTGTGGTGG |
|  | GK079-seqR | CCAGGAAGGCTTATTTCAGTC |
| *cusS* | GK080-F | GCGGCATGTTATTTTTACACTGGTTATAAAAGTTGCCGTTTGCTGAAGGA-GTGTAGGCTGGAGCTGCTTC |
|  | GK080-R | CAAAGTTGATTCAGACCGTGCGCGGCGTGGGTTACATGCTAGAGGTGCCG-CATATGAATATCCTCCTTAG |
|  | GK080-seqF | GAATTGTGACAGCTTTTTATCATT |
|  | GK080-seqR | GATACCAACGCCATTGATGT |

**b.**

| Primer ID | Sequence | Purpose |
| --- | --- | --- |
| GK073-F | GATCGGATCCAAGGGCGAGGAGGATAAC | Amplifying mCherry gene. |
| GK073-R | CTAGAAGCTTTTACTTGTACAGCTCGTCCATG | Amplifying mCherry gene. |
| GK074-F | CTAGGGATCCCATGGGAGTAACTGAATTTCC | Amplifying operon 1 promoter. |
| GK074-R | GATCGAGCTCCATGACCTGGTTATCTCCCT | Amplifying operon 1 promoter. |
| GK095-F | GCATGGGCCCTTCGCATGAATTCCATGACCTGGTTATCTCCCT | Amplifying operon 1 promoter and mCherry. |
| GK095-R | CTAGAAGCTTTTACTTGTACAGCTCGTCCATG | Amplifying operon 1 promoter and mCherry. |
| GK096-F | GATCGAATTCATGACGGAGTCACCGCAA | Amplifying *ybtA* gene. |
| GK096-R | GATCGGGCCCTTACATCCCGCGTTTAAAGGT | Amplifying *ybtA* gene. |
| C206SC209S-F | GACGCCAGTAGCCTCTCCAGCCACTTCAACCCGATGGAA | Making point mutations in *ybtA*. |
| C206SC209S-R | TTCCATCGGGTTGAAGTGGCTGGAGAGGCTACTGGCGTC | Making point mutations in *ybtA*. |
| GK084-F | GATCTCTAGAGGGAGTAACTGAATTTCCTGATGA | Amplifying operon 1 promoter for dual reporter. |
| GK084-R | GATCAAGCTTGACCTGGTTATCTCCCTGTGG | Amplifying operon 1 promoter for dual reporter. |

**c.**

| Primer ID | Sequence | Purpose |
| --- | --- | --- |
| ybtS mFwd | CCTCTTTCGCCTTATTATGCTC | Specific forward primer for ybtS. |
| ybtS mRev | CGCTCGTTTATGTTCCGTC | Specific reverse primer for ybtS. |
| GK007-F | CTGTTCGTTGCAGGAAGGTAT | Specific forward primer for ybtA. |
| GK007-R | AGTCCCTGAATCGCAAAGC | Specific reverse primer for ybtA. |
| GK009-F | CCAGTAAACAATCTTCCCGCT | Specific forward primer for fyuA. |
| GK009-R | GCATGTTGCCGCTATTTTC | Specific reverse primer for fyuA. |
| GK010-F | ATGAGATGGTTACACTGGTTTCG | Specific forward primer for irp2. |
| GK010-R | CGTTTCTTCTTCCGCGTTC | Specific reverse primer for irp2. |
| GK011-F | GTAAATACCATCCCCATGGTGA | Specific forward primer for gyrA. |
| GK011-R | ATTTCCGTATAACGCATTGCC | Specific reverse primer for gyrA. |
